# Readmission prediction after colorectal cancer surgery: A derivation and validation study

**DOI:** 10.1371/journal.pone.0287811

**Published:** 2023-06-29

**Authors:** Joel D’Souza, Timothy Eglinton, Frank Frizelle

**Affiliations:** Department of Surgery, Christchurch Hospital, University of Otago, Dunedin, New Zealand; University of L’Aquila, ITALY

## Abstract

**Background:**

Unplanned readmissions after colorectal cancer (CRC) surgery are common, expensive, and result from failure to progress in postoperative recovery. The context of their preventability and extent of predictability remains undefined. This study aimed to define the 30-day unplanned readmission (UR) rate after CRC surgery, identify risk factors, and develop a prediction model with external validation.

**Methods:**

Consecutive patients who underwent CRC surgery between 2012 and 2017 at Christchurch Hospital were retrospectively identified. The primary outcome was UR within 30 days after index discharge. Statistically significant risk factors were identified and incorporated into a predictive model. The model was then externally evaluated on a prospectively recruited dataset from 2018 to 2019.

**Results:**

Of the 701 patients identified, 15.1% were readmitted within 30 days of discharge. Stoma formation (OR 2.45, 95% CI 1.59–3.81), any postoperative complications (PoCs) (OR 2.27, 95% CI 1.48–3.52), high-grade PoCs (OR 2.52, 95% CI 1.18–5.11), and rectal cancer (OR 2.11, 95% CI 1.48–3.52) were statistically significant risk factors for UR. A clinical prediction model comprised of rectal cancer and high-grade PoCs predicted UR with an AUC of 0.64 and 0.62 on internal and external validation, respectively.

**Conclusions:**

URs after CRC surgery are predictable and occur within 2 weeks of discharge. They are driven by PoCs, most of which are of low severity and develop after discharge. Atleast 16% of readmissions are preventable by management in an outpatient setting with appropriate surgical expertise. Targeted outpatient follow-up within two weeks of discharge is therefore the most effective transitional-care strategy for prevention.

## Introduction

The readmission rate (RR) is a key performance indicator (KPI) that is widely used to evaluate the quality of hospital care delivered to a population [1–3]. Its well-validated association with the quality of healthcare, ease of calculation, and applicability to various patient cohorts underscores its clinical utility [1].

Unplanned readmissions (URs) have significant implications for both the patient and the healthcare system [4, 5]. They result from a failure in the continued progression of patient recovery toward their preoperative baseline. This is supported by their association with poor patient outcomes such as increased morbidity, poor quality of life, and increased mortality [4, 6]. URs are also associated with significant costs to the healthcare system, with an estimated economic impact of $17.4 billion per year in the US [7, 8]. In the context of preventability, this is an important consideration for rationing the limited healthcare resources in the Covid-19 era.

Previous comparative research on readmission has highlighted that medical readmissions are due to exacerbations of underlying comorbidity, whereas surgical readmissions comprise a small proportion of readmissions overall and are primarily due to complications arising from the index surgery [7, 8]. Therefore, surgical readmission is more amenable to risk-reduction interventions. When classified according to surgical specialty, colorectal surgery has one of the highest readmission rates (25%) [7, 9]. Successful readmission reduction interventions may therefore have a high yield in this specialty [6, 9–20].

Identification of robust risk factors for UR is critical for the development of readmission prediction models. The successful application of these models to high-risk individuals at discharge may facilitate targeted readmission-reduction interventions. Several studies have reported multiple risk factors for UR after colorectal surgery (CRS). However, this has not resulted in effective readmission prediction, primarily due to limitations arising from significant heterogeneity in this field of research [4, 8, 21, 22]. The major sources included variations in sample size, sample sources, methodology, definitions, time intervals to readmission, and non-standardized categorization of both demographic and clinical risk factors.

This derivation and validation study aimed to define the 30-day UR rate after colorectal cancer (CRC) surgery; identify clinically relevant risk factors for UR; develop a clinically applicable prediction model; internally and externally validate this prediction model; and define readmission characteristics according to timing, diagnoses, and severity. To address previous limitations arising from heterogeneity, a homogenous cohort of consecutive patients undergoing curative colorectal cancer surgery in an elective setting was used as the study sample.

## Materials and methods

### Patient selection

All patients who underwent elective CRC surgery between 2012 and 2017 at Christchurch Hospital were identified using the Binational Colorectal Cancer Audit (BCCA) database. The BCCA database was established by the Colorectal Surgical Society of Australia and New Zealand (CSSANZ). Consent to access de-identified data was obtained from the BCCA Operations Committee. Patients were excluded if they had recurrent colorectal cancer, stage 4 disease, non-curative surgery, or transanal excision of rectal tumours without a total mesorectal excision.

### Clinical variables

The patients’ medical records were retrospectively reviewed in parallel with Christchurch Hospital’s clinical portal for additional data quality assurance. Missing data from the BCCA database were obtained to avoid incomplete case exclusion. The relevant clinical variables were extracted and tabulated. These were classified as preoperative, operative, and postoperative variables.

Preoperative variables included age, sex, and American Society of Anesthesiologists (ASA) score. Operative variables included surgical approach, surgery type, stoma formation, stoma type, and tumor site. Rectal tumors were defined as those with the lowest margin of <15 cm above the anal verge, as measured on sagittal magnetic resonance imaging. Postoperative variables included the occurrence of any postoperative complications (PoCs), length of hospital stay (LOS), discharge disposition, and stage (American Joint Committee on Cancer staging system) [23]. Postoperative complications were classified by number and severity based on the Clavien-Dindo (CD) classification grade [24]. Postoperative complications grade-3 and above were further categorized as “high-grade PoCs”. The postoperative variable time-interval was defined as the day of surgery to discharge. All patients followed a standardised multimodal postoperative recovery pathway as per Enhanced Recovery after Surgery (ERAS).

### Primary outcome

The primary outcome was defined as any unplanned readmission within 30 days of the index discharge. The decision for readmission was a collaboration between the Emergency Department (ED) physicians and the acute general surgery team based on the need for inpatient general surgery care in the ongoing work-up and management of presenting patients. Patients discharged directly from the Emergency Department were excluded. Mortality was excluded from the readmission analysis. The secondary outcomes were the timing, diagnosis, duration, and severity of unplanned readmissions.

### Statistical analysis

Continuous variables are reported as medians with interquartile ranges (IQR) and categorical variables as whole numbers and percentages. Univariable analysis was performed to compare baseline characteristics between readmitted and non-readmitted patients. Appropriate statistical tests were selected based on variable type and parametricity. Multivariable logistic regression analysis was performed with stepwise backward selection, with a p-value greater than 0.05 used for exclusion of candidate variables. Regression coefficients for these model variables are reported as odds ratios (ORs) with 95% confidence intervals (CI). Multicollinearity between predictor variables was assessed by variance inflation factor (VIF), with scores of over 3 taken to indicate unacceptable collinearity. At least 15 events per candidate predictor were ensured to maintain statistical power for multivariable analysis, as recommended by the guidelines on prognostic factor research quality improvement [25]. A recent departmental audit demonstrated a readmission rate of 20%. A minimal sample size of 600 was hence required to power the multivariable analysis accordingly. Statistically significant predictor variables in the multivariable logistic regression analysis were incorporated into a predictive model. Statistical analysis was performed using R Studio software (version 3.5.2; www.rstudio.com).

### Ethics

Ethical approval was obtained from the University of Otago Human Ethics Committee (#H17/148) following confirmation from the National Health and Disability Ethics Committee as a minimal risk observational study. Locality approval was obtained by the Canterbury District Health Board (ID 17255). All data was stored in a secure hospital server with access given to the authors of this study. All subsequent analysis was performed on de-identified datasets. Patient privacy and confidentiality was maintained on all phases of the study.

### Model validation

The entire dataset was used to derive a predictive model and evaluate its performance. Internal validation was performed by bootstrapping to obtain a more precise estimate of model performance. The procedure involved repeating the modelling process with variable selection in 2000 bootstrap samples drawn with replacement from the original sample. This process quantified optimism secondary to overfitting and allowed for the adjustment of the regression coefficients and performance based on the estimated shrinkage factor. The model was then externally validated on a prospectively maintained sample set of patients based on the same inclusion criteria who underwent curative colorectal surgery between January 2018 and June 2019. The prospective validation design ensured consecutive patient selection and subsequent reduction of selection bias. Measures of model performance on this external sample set were then repeated and compared using similar statistical techniques.

### Model performance

The performance of the predictive model in the derivation cohort was assessed through calibration and discrimination. Calibration was measured by the degree of agreement between the observed events, and the models predicted-risk probabilities using a calibration plot. An intercept of 0 and a slope of 1 on a calibration plot indicate perfect calibration. Discrimination refers to the ability of a model to differentiate between individuals who do and do not experience the primary outcome. This was measured using the area under the receiver operating characteristic (ROC) curve, where a value of 0.50 represented chance and 1 represented perfect discrimination.

## Results

### Patient characteristics

A total of 701 patients underwent curative colorectal cancer surgery during the study period. The baseline characteristics, clinical variables, and frequencies are shown in Table 1. The median patient age was 72 years (IQR: 64–79 years). There were no significant differences in age, sex, ASA score, stage, or discharge disposition between the readmitted and non-readmitted patients. Anterior resection was the most common surgical procedure (44.1%) followed by colectomy (43.7%), APR (10.8%), and proctocolectomy (1.4%). The median LOS was 7 days (IQR: 5–10 days).

**Table 1:** Baseline patient characteristics.

Patient variables		Total (n = 701)	No UR (n = 595)	UR (n = 106)	Univariable Odd’s Ratio (95%I)	p-value
**Age (years)**	Median (IQR)	-	72 (63–79)	73 (64–78)	1.00 (0.98–1.02)	0.91
**Length of stay (days)**	Median (IQR)	-	6 (4 10)	8 (6–12)	1.05 (1.02–1.08)	<0.001
**Sex**	Female	317	269	48	Ref	0.99
	Male	384	326	58	1.00 (0.66–1.51)	
**ASA**	1	92	83	9	Ref	0.21
	2	374	318	56	1.62 (0.81–3.64)	
	3	220	180	40	2.05 (0.99–4.68)	
	4	15	14	1	0.66 (0.03–3.93)	
**Surgical entry**	Open	377	312	65	Ref	0.047
	Laparoscopic	295	261	34	0.63 (0.40–0.97)	
	Conversion to open	29	22	7	1.53 (0.58–3.56)	
**Procedure type**	Colectomy	306	275	31	Ref	<0.001
	Proctocolectomy	10	8	2	2.22 (0.33–9.34)	
	Anterior Resection	309	258	51	1.75 (1.09–2.85)	
	APR	76	54	22	3.61 (1.93–6.70)	
**Stoma**	No	426	385	41	Ref	<0.001
	Yes	275	210	65	2.91 (1.91–4.48)	
**Stoma type**	No Stoma	426	385	41	Ref	<0.001
	End Colostomy	93	71	22	2.91 (1.62–5.14)	
	End Ileostomy	11	8	3	3.52 (0.75–12.70)	
	Loop Colostomy	4	2	2	9.39 (1.10–80.00)	
	Loop Ileostomy	167	129	38	2.77 (1.70–4.49)	
**Tumour Site**	Colon	430	385	45	Ref	<0.001
	Rectum	271	210	61	2.50 (1.65–3.83)	
**AJCC Stage**	Stage 0	44	34	10	Ref	0.39
	Stage 1	157	135	22	0.55 (0.24–1.32)	
	Stage 2	255	221	34	0.52 (0.24–1.20)	
	Stage 3	245	205	40	0.66 (0.31–1.51)	
**Any postoperative complications**	No	418	376	42	Ref	<0.001
	Yes	283	219	64	2.62 (1.71–4.02)	
**High-grade postoperative complications**	No	662	568	94	Ref	0.01
	Yes	39	27	12	2.69 (1.27–5.37)	
**Clavien-Dindo postoperative complication grade**	0	418	376	42	Ref`	<0.001
	1	69	55	14	3.76 (1.04–12.00)	
	2	175	137	38	0.90 (0.26–2.91)	
	3a	13	10	3	1.32 (0.49–3.51)	
	3b	24	14	7	0.78 (0.32–1.85)	
	4	5	3	2	0.95 (0.34–2.25)	
	5	0	0	0	-	
**Number of postoperative complications**	0	446	397	49	Ref	<0.001
	1	180	139	41	2.39 (1.51–3.77)	
	2	51	38	13	2.77 (1.34–5.45)	
	3+	24	21	3	1.16 (0.27–3.51)	
**Discharge disposition**	Home	634	538	96	Ref	0.32
	Rehab	57	50	7	0.78 (0.32–1.67)	
	HLC (baseline)	1	1	0	-	
	RHLC (baseline)	8	5	3	-	
	RHLC (new)	1	1	0	-	

### Unplanned readmission rate

**Fig 1:**
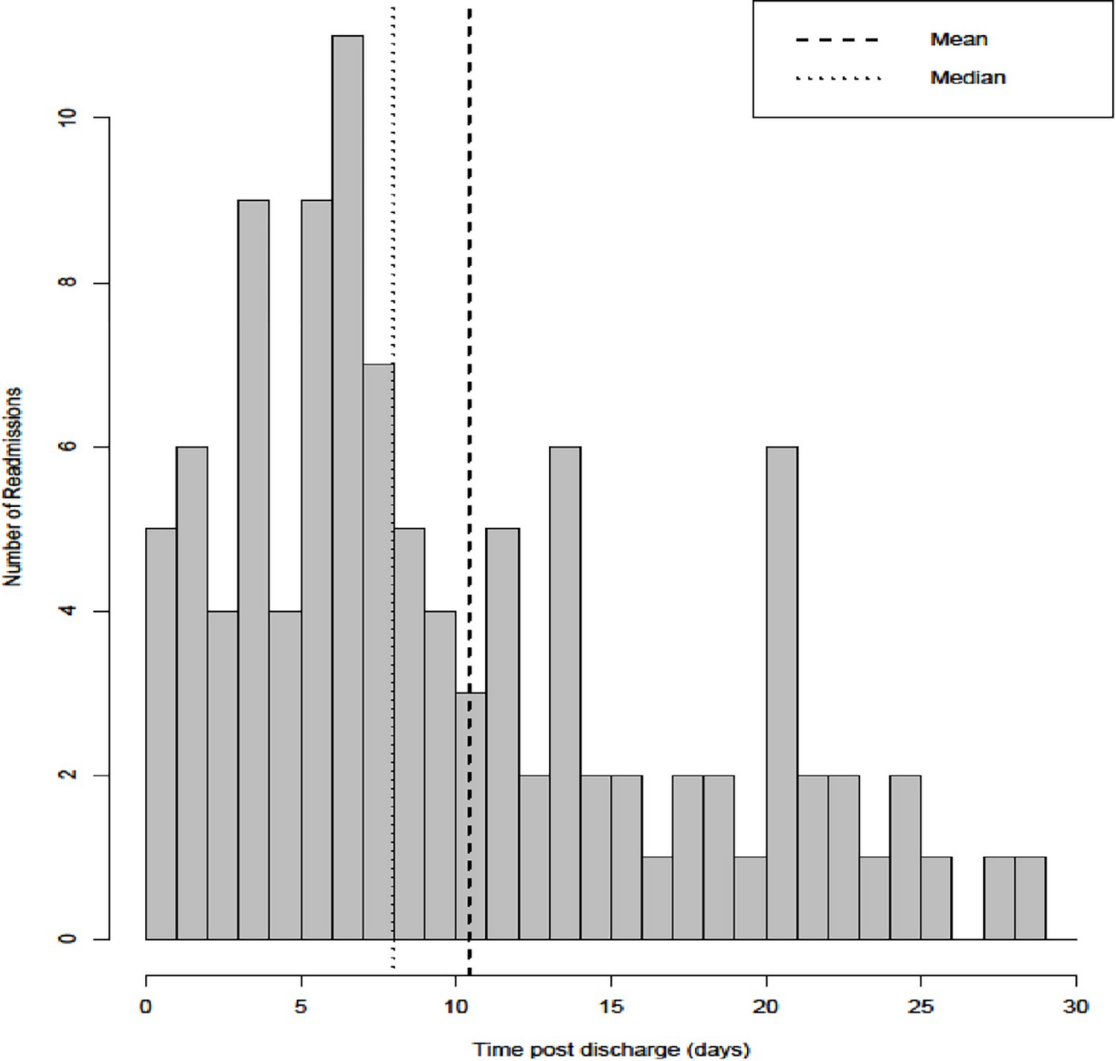
Time to unplanned readmission (days).

Of the 701 patients discharged alive, 106 were readmitted within 30 days of the index discharge, giving an unplanned readmission rate of 15.1%. The timing of UR in relation to the index discharge is shown in Fig 1. The median time to UR after the index discharge was 8 days (IQR: 5–14 days). Approximately 75% of the URs (n = 80) occurred within two weeks of discharge, of which 45% occurred within one week after discharge (n = 48). The median duration of the URs was three days (IQR: 1–6 days).

### Unplanned readmission characteristics

The readmission characteristics are shown in Table 2. The three most common readmission diagnoses were wound infection (27%), intra-abdominal collection (22%), and bowel obstruction or ileus (14%). The corresponding time to readmission and LOS for each diagnosis are listed in Table 2. All readmissions were for diagnoses related to the index surgery. When graded according to severity, 75% of unplanned readmissions were CD grade-1 and grade-2, as shown in Table 3.

**Table 2:** Unplanned readmission characteristics.

Readmission Diagnosis	Number of Readmissions	Median day of presentation post discharge, (IQR)	Median Readmission LOS (days), (IQR)	CT Imaging on Readmission	Similar pre-discharge PoCs	Diagnosis related to Index Surgery
Wound infection/ dehiscence	29	9 (6–14)	1 (1–2)	12	5	29
Intra-abdominal Collection/anastomotic leak	23	7 (4–14)	7 (4–10)	21	7	23
Bowel Obstruction	15	6 (3–10)	4 (3–6)	11	5	15
Urosepsis	8	12 (6–19)	3.5 (3–4)	7	1	8
Postoperative pain	7	18 (8–20)	2 (0–3)	3	0	7
Stoma related	6	9 (7–12)	5 (2–9)	2	0	6
Chest pain/ arrhythmia	6	-	-	2	2	6
Pneumonia	4	-	-	1	1	4
Colitis/Enteritis	3	-	-	2	0	3
PR bleed	2	-	-	1	0	2
Hyperglycaemia	1	-	-	0	0	1
CVA	1	-	-	1	0	1
Syncope	1	-	-	0	0	1
**Total**	106	8 (5–14)	3 (1–6)	63 (59%)	21 (20%)	106 (100%)

**Table 3:** Unplanned readmission severity (Clavien-Dindo).

Clavien-Dindo grade	Number of Unplanned Readmissions
Grade 1	32
Grade 2	48
Grade 3a	14
Grade 3b	11
Grade 4	0
Grade 5	1
Total	106

### Risk factors for unplanned readmission

Clinical variables and their corresponding univariable and multivariable odd’s ratios are shown in Table 4. Length of Stay, surgical entry, surgery type, stoma, tumor site, and PoCs were found to be significant in the univariable analysis. The tumor site and stoma variables showed significant collinearity (VIF = 3.22). This collinearity was addressed by substituting the corresponding collinear variables in the analysis.

**Table 4:** Multivariable analysis.

		Unadjusted Odd’s Ratio (95%CI)	p-value	Adjusted Odd’s Ratio (95% CI)	p-value
**Length of stay (days)**	Median (IQR)	1.05 (1.02–1.08)	0.001	1.00 (0.96–1.04)	0.93
**ASA Score**	1	Ref	0.213	Ref	0.23
	2	1.62 (0.81–3.64)		1.52 (0.74–3.46)	
	3	2.05 (0.99–4.68)		1.95 (0.92–456)	
	4	0.66 (0.03–3.93)		0.64 (0.33–3.96)	
**Surgical Entry**	Open	Ref	0.047	Ref	0.22
	Laparoscopic	0.63 (0.40–0.97)		0.90 (0.56–1.44)	
	Conversion to open.	1.53 (0.58–3.56)		2.26 (0.84–5.57)	
**Procedure Type**	Colectomy	Ref	<0.001	Ref	0.43
	Proctocolectomy	2.26 (0.33–9.38)		1.08 (0.15–5.19)	
	Anterior Resection	1.77 (1.10–2.87)		1.23(0.68–2.22)	
	APR	3.62 (1.94–6.73)		1.81 (0.80–4.10)	
**Stoma**	No	Ref	0.007	Ref	<0.001
	Yes	2.89 (1.89–4.44)		2.45 (1.59–3.81)	
**Tumour Site**	Colon	Ref	<0.001	Ref	<0.001
	Rectum	2.50 (1.65–3.83)		2.11 (1.48–3.52)	
**Any postoperative complications**	No	Ref	<0.001	Ref	<0.001
	Yes	2.60 (1.71–3.99)		2.27 (1.37–3.26)	
**High-grade postoperative complications**	No	Ref	0.005	2.52 (1.18–5.11)	0.04
	Yes	2.69 (1.27–5.37)			

On multivariable logistic regression, the occurrence of PoCs (OR 2.27, 95% CI 1.37–3.26), stoma (OR 2.45, 95% CI 1.59–3.81), and rectal cancer (OR 2.11, 95% CI 1.48–3.52) remained statistically significant. High-grade PoCs remained a statistically significant variable when used as a candidate predictor instead of PoCs (OR 2.52, 95% CI 1.18–5.11).

### Prediction of unplanned readmission

To address collinearity, tumor site was chosen as a predictor variable instead of stoma. Two simple clinically applicable models for predicting unplanned readmission were assessed using the following predictor variables: tumor site, PoCs, and high-grade PoCs.

Model _A_ consisted of two variables: tumor site and PoCs. Similarly, model _B_ consisted of two variables: tumor site and high-grade PoCs.

**Table:** 

***Model***_***A***_	=	***Tumour Site***	+	***PoCs***
***Model***_***B***_	=	***Tumour Site***	+	***High-grade PoCs***

### Internal validation

**Fig 2:**
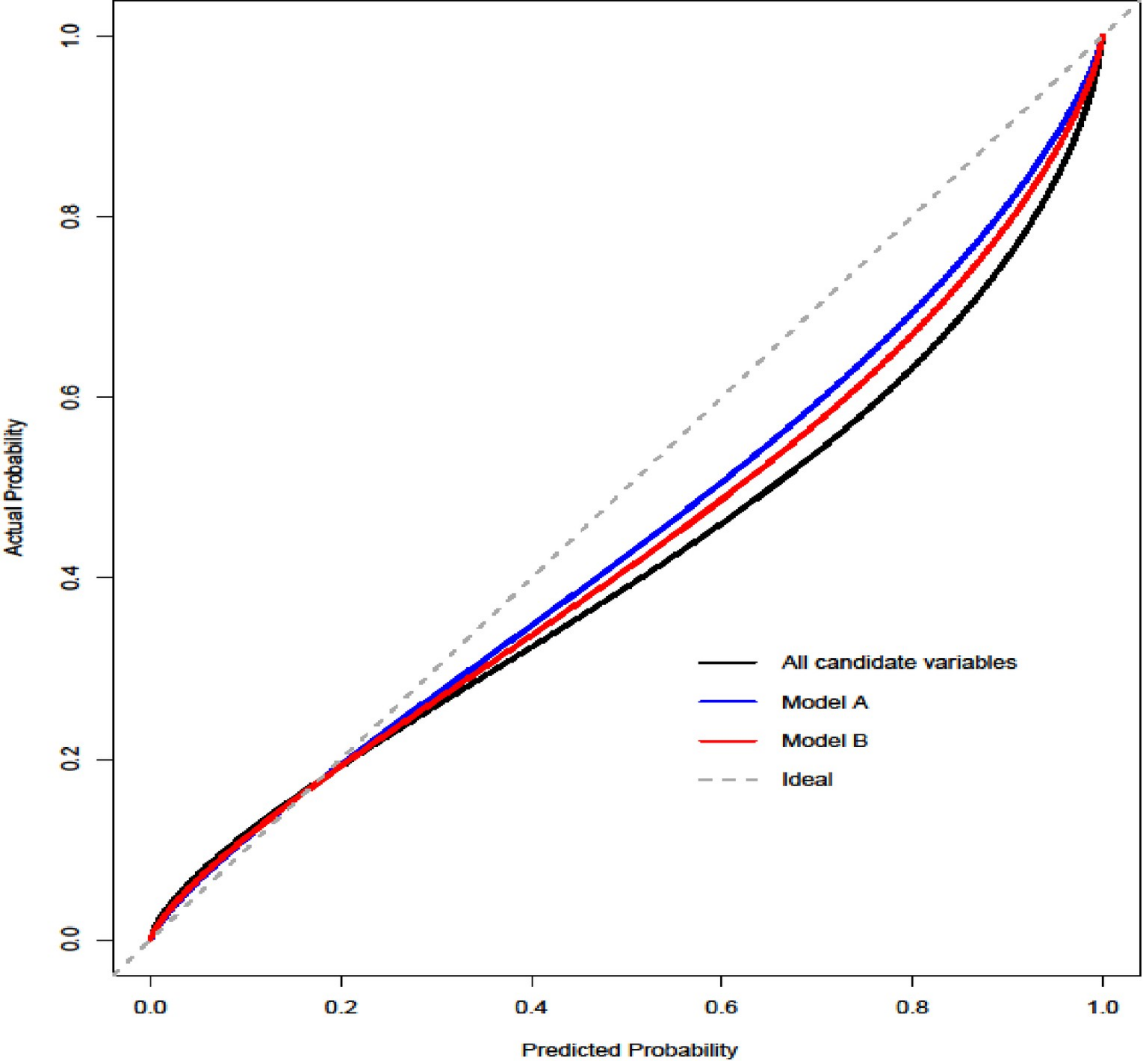
Calibration curves of model A and model B.

**Fig 3:**
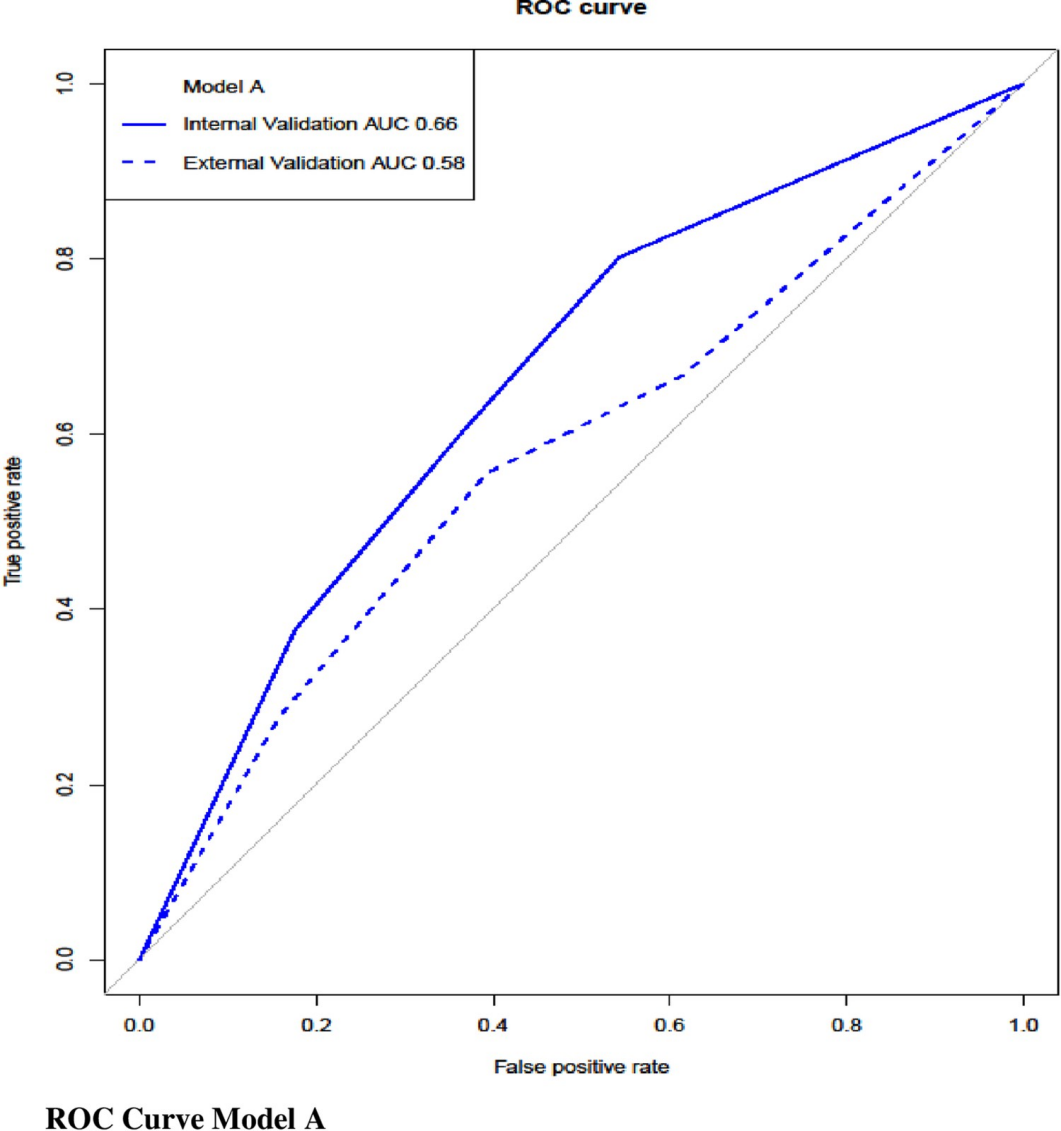
ROC curve model A.

**Fig 4:**
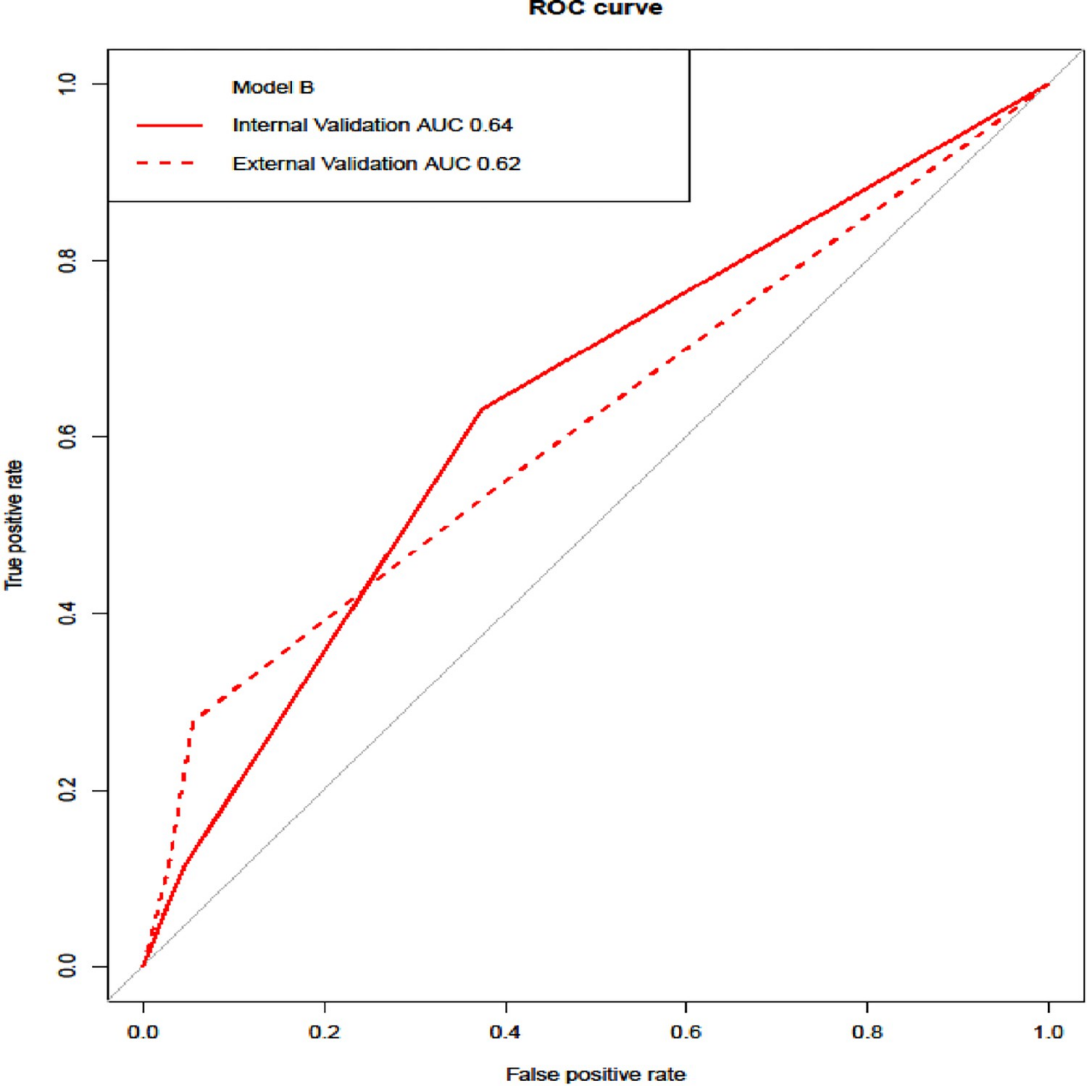
ROC curve model B.

Both models were internally validated on the derivation sample set using bootstrapping with replacement as previously described. A model consisting of all the significant variables in the univariable analysis was used for comparison. Comparative model performance was demonstrated using calibration plots and ROC curves. As shown in the calibration plot in Fig 2, both models overestimated the predicted probability of readmission, although model _B_ was closer to the ideal slope of 1. Discrimination for both models on internal validation is shown by the ROC curves in Figs 3 and 4. The AUC for model _A_ and model _B_ were 0.66 and 0.64, respectively.

#### External validation

Both the models were externally validated on a prospective sample set of 156 patients. The overall unplanned readmission rate was 11.5% (n = 18). Baseline characteristics of the validation cohort are presented in the supporting information (S1 Table in S1 File). The discrimination ability of both models on external validation are shown on the ROC curves in Figs 3 and 4, in addition to their respective internal validation AUC. The AUC for Model _A_ decreased from 0.66 to 0.58, compared with Model _B_, which decreased from 0.64 to 0.62. Therefore, Model _B_ was demonstrated to be a superior predictive model.

### Regression model _B_

The resulting logit regression model (Model _B_) after internal validation with bootstrapping can be expressed as:

$$log\left(\frac{P_{readmission}}{{1-P}_{readmission}}\right)=-2.21+0.90\;(Tumour\;site="Rectum")+0.92\;("Highgrade\;PoCs")$$

where P_readmission_ denotes the probability that a patient is readmitted within 30 days of index discharge. The tumor site variable had a value of “1” for rectal tumors and “0” for colonic tumors. Similarly, the CD3^+^ variable had a value of “1” for the occurrence of high-grade PoCs and “0” for no high-grade PoCs.

Alternatively, the model can be represented according to the following equation to calculate the probability of readmission.


$$P_{readmission}=\frac{e^{-2.21+0.90\;(Tumour\;Site=Rectum)+0.92\;(High-grade\;PoCs)}}{{1+e\;}^{-2.21+0.90\;(Tumour\;site=Rectum)+0.85\;(High-gradePoCs)}}$$

## Discussion

The findings of this retrospective study demonstrated that 15% of the patients who electively underwent curative CRC surgery were readmitted within 30 days of index discharge, and all were for complications related to index surgery. The three most common readmission diagnoses were wound infection, intra-abdominal sepsis, and bowel obstruction. Approximately 75% of the URs occurred within 2 weeks of index discharge and were of low severity, as reflected by their CD grade and median LOS of 3 days. The three main risk factors for UR on multivariable analysis were stoma formation, rectal cancer, and the development of any PoCs before discharge.

Stoma formation was a significant risk factor for UR (adjusted OR 2.45, 95% CI 1.59–3.81). This is consistent with the findings of other systematic reviews of colorectal surgery readmissions [21, 22]. In addition, the underlying correlation between stoma formation and readmission in these studies was attributed to stoma-related complications such as electrolyte abnormalities and renal failure. In contrast, these stoma-related complications accounted for only 5% of the readmissions in this study. Similarly, patients with rectal tumors were at an increased risk of UR than those with colonic tumors (adjusted OR 2.11, 95% CI 1.48–3.52). This finding was supported by two studies that assessed this variable in relation to readmission and attributed it to the increased risk of morbidity associated with rectal dissection [13, 26]. In line with this consideration, of the 271 rectal tumor resections in our study, >90 also had a stoma. The remaining stoma formation cases in our study (n = 28) were distal colon cancers that required rectal mobilization to obtain oncological margins, physiological intraoperative decompensation, positive anastomosis leak tests, and patient choice. This explains the high collinearity between rectal tumors and stoma formation in our analysis. Clinically relevant baseline characteristics of the rectal tumors from the overall cohort are shown in the supporting information (S2 Table in S1 File). Of 271 rectal tumor resections, > 90% required intraoperative stoma formation. Fifty-five percent of these cases developed a postoperative complication, and 25% resulted in unplanned readmission, in comparison to the overall unplanned readmission incidence of 15%. Based on these findings, rectal tumor and stoma variables represented cases associated with increased morbidity. This increased morbidity led to the development of PoCs, a proportion of which resulted in unplanned readmission.

In further support of this, the development of any PoC was a significant risk factor for UR (adjusted OR 2.27, 95% CI 1.37–3.26). Studies outside the scope of this review across a range of surgical specialties have also reported an association between PoCs and UR [4, 9, 20, 22, 27]. In addition, a dose-response relationship between the number of PoCs and the risk of UR has been reported in studies not limited to colorectal surgery [27–31]. Further delineation of how the development of PoCs modulates the risk of UR was limited by the lack of reporting of PoC characteristics and heterogeneity from non-standardized definitions. To address this limitation, this study defined postoperative complications and their characteristics as shown in Tables 1, 2, and 5. A dose-response relationship was observed between the number of PoCs and the risk of UR, reaffirming the findings of previous studies in the colorectal cancer surgery setting. This trend lost statistical significance in patient with three or more PoCs, owing to the small number of these patients (<3.4%). When classified by severity, high-grade PoCs were a significant risk factor for UR.

**Table 5:** Postoperative complications.

Complication Type	Number Overall	No Readmission	Readmission
Postoperative Ileus	115	92	23
Cardiorespiratory	67	52	15
Urosepsis	45	36	9
Postoperative Haemorrhage	33	27	6
Urinary Retention	31	28	3
Intra-abdominal Sepsis	31	21	10
Wound infection/dehiscence	13	11	2
Enteritis	4	4	0
Delirium	3	1	2
Venous Thromboembolism	2	0	2
Cerebrovascular accident	2	2	0
Stoma complications	2	1	1
Postoperative Hypotension	2	1	1
Acute Renal Impairment	2	1	1
Thrombophlebitis	1	1	0
Anaphylaxis	1	1	0
Orchiditis	1	1	0
Postoperative Hernia	1	1	0
Sepsis NOS	1	0	1
**Total**	356	280 (78.7%)	76 (21.3%)

The results of this study also defined the timing of PoCs in relation to the index readmission. All readmissions were for PoCs related to the index surgery. Of the 106 readmissions, 21 matched similar PoCs that occurred before index discharge (Table 2). This demonstrated that 80% of readmissions were new postoperative complications that were either clinically silent or absent at the time of discharge and, therefore, developed after discharge in the community. This further supports the premise that URs after elective colorectal cancer surgery serve as surrogates for PoCs that develop after discharge.

Two prediction models were evaluated using statistically significant variables with the goal of being simple and clinically applicable. Both models used only two predictor variables, with model _B_ demonstrating better discriminatory ability on external validation (AUC 0.62). Given the collinearity between the variables of tumor site and stoma, the former was chosen given the preoperative availability of this information. Two hundred eighty-three patients developed PoCs compared to 39 patients who developed high-grade PoCs. Therefore, the high-grade PoC variable provides a more precise target for patients at risk of UR than the PoCs variable.

The percentage of readmissions deemed to be preventable varied significantly in the literature, ranging from 5% to 79% [1, 14, 27, 32–34]. This wide range is due to subjective estimation of preventability based on administrative datasets. The findings of this study show that the underlying aetiology of unplanned readmissions are postoperative complications, the majority of which develop after discharge. Postoperative complications remain a routine part of surgical care despite all preventative measures taken to minimize their occurrence. These measures include appropriate patient selection, optimization of preoperative comorbidities, fastidious surgical techniques, and appropriate postoperative protocols [35, 36]. Despite patients following ERAS in this study, compliance was not measured due to limitations from the retrospective design. Compliance to ERAS has not been shown to influence readmission and is therefore unlikely to have influenced the primary endpoint of this study [37].

The clinical relevance of readmission prevention is blurred when considered solely in terms of their underlying aetiology. However, if a proportion of them can be successfully managed in the outpatient despite their underlying aetiology, they can then be deemed preventable, and clinically relevant. Wound infections that were diagnosed clinically without imaging comprised 16% of overall readmissions. This number reflects the minimum percentage of readmissions that could be preventable through outpatient management. This number is likely to be greater given that 41$ of all readmissions did not require diagnostic imaging, and 75% were of low severity (CD grade 1 and 2). Given the need for surgical expertise in their assessment and management, the interventional strategy of targeted outpatient follow-up within a two-weeks of index discharge is the transitional care strategy supported by this study for readmission prevention. The findings of this study support the idea that URs after CRC surgery are preventable, but the true extent of prevention through outpatient management is difficult to delineate due to its retrospective design and subjective nature of defining preventability. Further studies on readmission characteristics after implementing this transitional care strategy are required to further quantify this preventability.

## Conclusions

The results of this study demonstrated that URs after colorectal cancer surgery are predictable. A proportion of these URs can be managed in the outpatient setting thus preventing and reducing readmissions. Patients with rectal cancer and those who developed high-grade PoCs during their index hospital stay had the highest risk of readmission and within two weeks of index discharge. Interventions to reduce readmissions, such as early clinical follow-up, may therefore be targeted toward these patients within this time interval. This strategy may prevent hospital readmissions though outpatient clinic management. The premise that readmissions represent a waste of healthcare resources is not supported by this study. While they represent a significant cost to the healthcare system, the need for surgical expertise in their management for the benefit of the patient justifies this expense.

### Limitations

This study has several limitations. Its retrospective methodology makes it susceptible to bias from known and unknown confounders. The small sample size for some predictor variable categories may have allowed for the possibility of a type two error. The model development process was conducted according to the TRIPOD guidelines [38]. Differences were observed in the primary event rates of the validation dataset (11%, n = 18). Limited empirical evidence dictates a validation sample size of a minimum of 100 events and 100 non-events, with 250 events being preferable. Therefore, further validation studies are required to evaluate the formulated prediction models in this study.

## Supporting information

S1 Checklist STROBE checklist. (DOCX)

S1 File Contains all the supporting tables. (DOCX)

S1 Dataset (CSV)

S2 Dataset (CSV)
