# Supplementary material for: Readmission prediction after colorectal cancer surgery: A derivation and validation study
Source: PLoS One. 2023 Jun 29;18(6):e0287811. doi: 10.1371/journal.pone.0287811 (PMC10309978; doi:10.1371/journal.pone.0287811)
Supplement: S1 File — (DOCX) [file pone.0287811.s002.docx]

**S-Table 1: External validation sample baseline characteristics**

| **Patient Variables** |  | **Total Number**  **(n = 156)** | **No Readmission**  **(n = 138)** | **Readmission**  **(n = 18)** |
| --- | --- | --- | --- | --- |
| **Age (Years)** | Median  (IQR) | - | 75  (65 - 80) | 71  (66 - 80) |
| **LOS (days)** | Median  (IQR) | - | 6  (4 - 10) | 7  (5 - 12) |
| **Sex** | Female  Male | 73  83 | 65  73 | 8  10 |
| **ASA** | 1  2  3  4 | 9  83  63  1 | 7  76  54  1 | 2  7  9  0 |
| **Surgical Entry** | Open  Laparoscopic  Conversion to Open | 55  98  3 | 50  85  3 | 5  13  0 |
| **Procedure** | Colectomy  Proctocolectomy  Anterior Resection  APR | 76  1  65  14 | 68  1  57  12 | 8  0  8  2 |
| **Stoma** | No  Yes | 100  56 | 89  49 | 11  7 |
| **Stoma Type** | No Stoma  End Colostomy  End Ileostomy  Loop Colostomy  Loop Ileostomy | 100  17  4  0  35 | 89  15  4  0  30 | 11  2  0  0  5 |
| **Tumour Site** | Colon  Rectum | 98  59 | 87  51 | 11  7 |
| **AJCC Stage** | Stage 0  Stage 1  Stage 2  Stage 3 | 15  37  56  48 | 13  33  49  43 | 2  4  7  5 |
| **Any Postoperative Complication** | No  Yes | 92  64 | 84  51 | 8  10 |
| **Clavien-Dindo 3+ postoperative complications** | No  Yes | 145  11 | 132  6 | 13  5 |
| **Clavien-Dindo classification grade** | 0  1  2  3a  3b  4  5 | 92  22  31  3  5  3  0 | 84  18  30  1  2  3  0 | 8  4  1  2  3  0  0 |
| **Discharge Disposition** | Home  Rehab | 144  12 | 128  10 | 16  2 |

**S-Table 2: Rectal tumour characteristics**

| **Patient variables** | | **Total**  **(n = 271)** | **No Stoma**  **(n = 24)** | **Stoma**  **(n = 247)** | **Univariable Odd’s Ratio**  **(95%I)** | **p-value** |
| --- | --- | --- | --- | --- | --- | --- |
| **Age (years)** | Median  (IQR) | - | 70  (66-74) | 69  (60 - 75) | 0.99  (0.95 – 1.02) | 0.80 |
| **Length of stay (days)** | Median  (IQR) | - | 5  (4 - 8) | 10  (7 - 14) | 1.41  (1.21 – 1.71) | <0.001 |
| **Sex** | Female  Male | 92  179 | 9  15 | 83  164 | Ref  1.18 (0.48 – 2.78) | 0.70 |
| **ASA** | 1  2  3  4 | 36  156  76  3 | 3  13  8  0 | 33  143  68  3 | Ref  1.00 (0.22 – 3.32)  0.77 (0.61 – 2.87)  - | 0.89 |
| **Any postoperative complications** | No  Yes | 126  145 | 16  8 | 110  137 | Ref  2.49 (1.05 – 6.34) | 0.04 |
| **CD3+ postoperative complications** | No  Yes | 252  19 | 24  0 | 228  19 | - | 0.04 |
| **Clavien-Dindo postoperative complication grade** | 0  1  2  3a  3b  4  5 | 126  36  90  7  8  4  0 | 16  4  4  0  0  0  0 | 110  32  86  7  8  4  0 | - | 0.01 |
| **Readmission** | No  Yes | 210  61 | 24  0 | 186  61 | - | 0.006 |
